# Registration-free workflow for electromagnetic and optical navigation in orbital and craniofacial surgery

**DOI:** 10.1038/s41598-021-97706-5

**Published:** 2021-09-10

**Authors:** R. Schreurs, F. Baan, C. Klop, L. Dubois, L. F. M. Beenen, P. E. M. H. Habets, A. G. Becking, T. J. J. Maal

**Affiliations:** 1 Department of Oral and Maxillofacial Surgery, Amsterdam UMC Location AMC and Academic Centre for Dentistry Amsterdam (ACTA), University of Amsterdam, Meibergdreef 9, 1105 AZ Amsterdam, The Netherlands; 2 Radboudumc 3DLab The Netherlands, Radboud University Medical Center, Radboud Institute for Health Sciences, Geert Grooteplein Zuid 10, 6525 GA Nijmegen, The Netherlands; 3 Department of Radiology and Nuclear Medicine, Amsterdam UMC Location AMC, University of Amsterdam, Meibergdreef 9, 1105 AZ Amsterdam, The Netherlands; 4 Department of Medical Biology, Section Clinical Anatomy and Embryology, Amsterdam UMC Location AMC, University of Amsterdam, Meibergdreef 9, 1105 AZ Amsterdam, The Netherlands

**Keywords:** Biomedical engineering, Health care

## Abstract

The accuracy of intra-operative navigation is largely dependent on the intra-operative registration procedure. Next to accuracy, important factors to consider for the registration procedure are invasiveness, time consumption, logistical demands, user-dependency, compatibility and radiation exposure. In this study, a workflow is presented that eliminates the need for a registration procedure altogether: registration-free navigation. In the workflow, the maxillary dental model is fused to the pre-operative imaging data using commercially available virtual planning software. A virtual Dynamic Reference Frame on a splint is designed on the patient’s fused maxillary dentition: during surgery, the splint containing the reference frame is positioned on the patient’s dentition. This alleviates the need for any registration procedure, since the position of the reference frame is known from the design. The accuracy of the workflow was evaluated in a cadaver set-up, and compared to bone-anchored fiducial, virtual splint and surface-based registration. The results showed that accuracy of the workflow was greatly dependent on tracking technique used: the workflow was the most accurate with electromagnetic tracking, but the least accurate with optical tracking. Although this method offers a time-efficient, non-invasive, radiation-free automatic alternative for registration, clinical implementation is hampered by the unexplained differences in accuracy between tracking techniques.

## Introduction

Accurate intra-operative registration is the cornerstone to acquire reliable positional information in intra-operative navigation^1–9^. The ideal registration method would be non-invasive, little time consuming, not logistically challenging, automatic and thus not user dependent, usable in every patient, compatible with each tracking technique (optical and electromagnetic (EM)), not exposing the patient to additional radiation and, most of all, accurate.

Currently, several registration concepts exist in craniofacial surgery: fiducial markers, splints, or a combination of the two may be used in point-based registration^3,5,7,10,11^. Surface-based registration may be accomplished through touch or laser surface scanning. Next to specific drawbacks regarding accuracy, invasiveness and usability, each of these methods requires user interaction. The result of the registration process will be user dependent to some degree. Automatic Image Registration overcomes the user-dependency issue: intra-operative imaging is acquired with the Dynamic Reference Frame (DRF) in place^7,10,12^. If a virtual planning is made on the pre-operative image set, image fusion allows integration of the intra-operative registration scan in the virtual surgical planning. While the user-dependency drawback is eliminated, issues regarding radiation exposure and extended operation time remain.

In this study, a registration-free dental splint-based method is proposed that eliminates user dependency and does not require acquisition of additional intra-operative imaging. The methodology of registration-free navigation is outlined and the accuracy is compared to bone-anchored maxillary fiducials (optical and EM) and surface-based registration (EM).

## Methods

### Preparations

**Figure 1:**
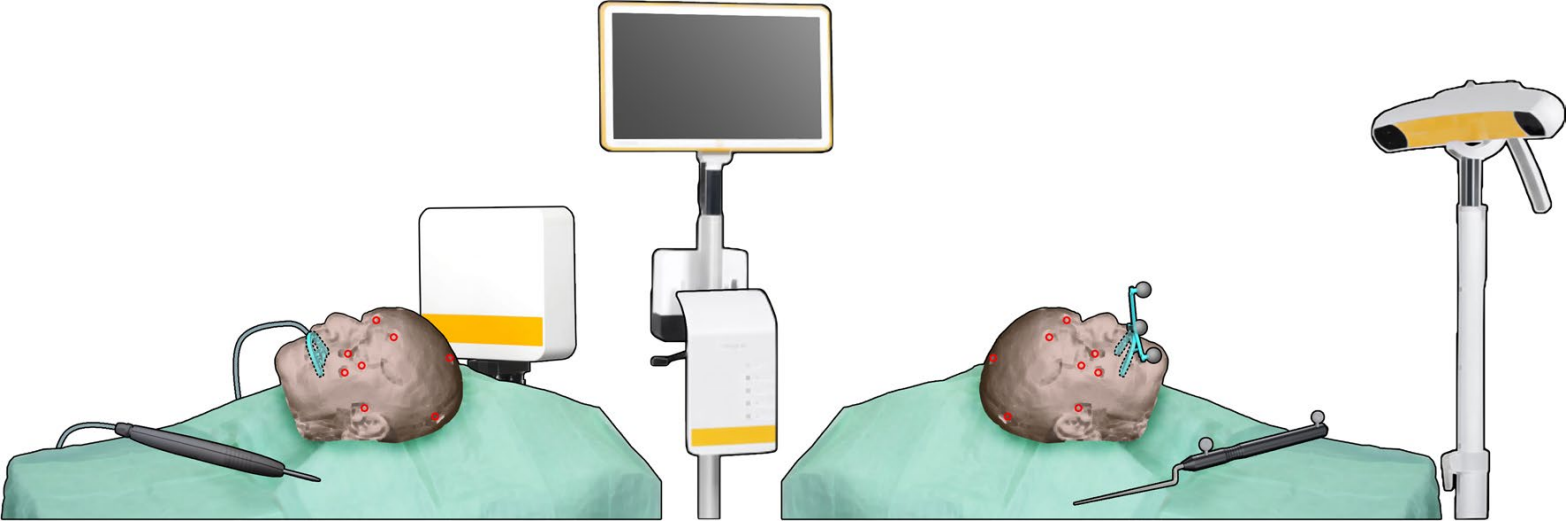
Schematic overview of the experimental set-up. The set-up on the left is the electromagnetic tracking set-up, with the field generator positioned lateral to the cadaver head. The DRF is positioned in the holder on the splint (detailed design in Fig. 2f). The set-up visualized on the right is the optical set-up, with the splint, with DRF attached, in place. The positions of the Allen target screws are indicated by the red circles.

Five dentulous cadaver heads were obtained through the body donation program from the Department of Medical Biology, Section Clinical Anatomy and Embryology of the Amsterdam UMC (location AMC). The bodies from which the samples were taken were donated to science in accordance with Dutch legislation and the regulations of the medical ethical committee of the Amsterdam UMC. The experimental protocol was approved by the review committee of Medical Biology, Section Clinical Anatomy and Embryology (ref. 2018–087). All methods were performed in accordance with the relevant guidelines and regulations. The dental status (maxilla) of the cadavers is shown in Table 1. The fixated cadaver heads were equipped with five titanium screws (1.5 × 5.0 mm maxDrive screws, KLS Martin, Tuttlingen, Germany) on the maxilla for bone-anchored fiducial registration, and fourteen Poly-Ether-Ether-Ketone (PEEK) Allen screws to serve as target positions at the following anatomical landmarks: orbital rim (bilaterally), zygomatic prominence (bilaterally), lateral orbital wall (bilaterally), porion (bilaterally), nasion, frontal bone (bilaterally), cranium and occipital bone (bilaterally). A Computed Tomography (CT) scan was acquired and imported in the Origin/Brainlab environment (iPlan version 3.0.6, Brainlab AG, Munich, Germany), a digital landmark was indicated on the Allen screw positions. The coordinates of these landmarks were used as the ground truth in the Target Registration Error (TRE) quantification^2,13,14^. The experimental set-up is visualized in Fig. 1.

**Table 1:** Overview of the status of the maxillary dentition of the cadavers.

Cadaver	Decayed	Missing	Span	Filled	Metal filling	Composite filling
1	–	–	17–27	12	4	8
2	8	5	17–23	–	–	–
3	–	9	14–23	1	1	–
4	–	–	17–27	1	–	1
5	2	4	17–27	2	2	–

The DMFT score was provided, as well as the filling material and the most distal element present on either side.

### Conventional methods

#### Bone-anchored fiducial registration and soft-tissue registration TRE assessment

The Kick navigation system (Brainlab AG, Munich, Germany) was used for all measurements, since this system is compatible with optical and electromagnetic tracking. Brainlab’s craniomaxillofacial module (CMF) was used for optical navigation; soft-tissue registration was not available in this module. The ENT module was used for all EM measurements. The Dynamic Reference Frame (DRF) corresponding to the tracking method was fixated to the lateral skull. Bone-anchored fiducial registration was performed by indicating the five maxillary screws that had been inserted; the virtual registration points had been indicated in the Brainlab environment. Each observer (RS, FB) performed five repetitions of registration with bone-anchored fiducials with optical tracking and five repetitions of bone-anchored fiducial registration with electromagnetic tracking. Soft-tissue registration with electromagnetic tracking was performed according to the instructions provided by the system. This registration was repeated five times by both observers as well. After each registration, the navigation instrument was positioned at the Allen screws (target positions) and the coordinates were stored through the *Acquire*-functionality.

### Experimental method

#### Registration-free workflow theoretical background

Mathematically, the registration procedure links the patient space (physical space) and image space. The pose of the DRF ($${T}_{DRF}$$) on the patient is established in the image space ($${T}_{reg}$$). The more accurate the registration procedure is performed, the more accurate the virtual position of the DRF in the image space ($${T}_{reg}$$) resembles the actual position of the DRF on the patient ($${T}_{DRF}$$). After a registration is completed, $${T}_{reg}$$ is stored by the navigation system. After registration, the position of the pointer’s tip in the patient space (translation component $${t}_{PTR}$$ of pointer pose $${T}_{PTR}$$) can be expressed as coordinates ($$c)$$ in the image space. In TRE measurements, the pointer is positioned at a predefined location in the patient space (i.e., the PEEK screw heads); the measured position of the pointer in the image space ($$c$$) is compared to the actual position of the target in the image space ($$l$$). An overview of the transformations involved in the process and their underlying connections is provided in Appendix I; a schematic drawing of the registration-free approach is shown in Appendix I Fig. 1. The hypothesis behind the registration-free approach is that the DRF is inserted in a known pose in the patient space. The pose of the DRF in the image space can be determined preoperatively, rendering any intra-operative registration mute.

#### Registration-free workflow practical implementation

**Figure 2:**
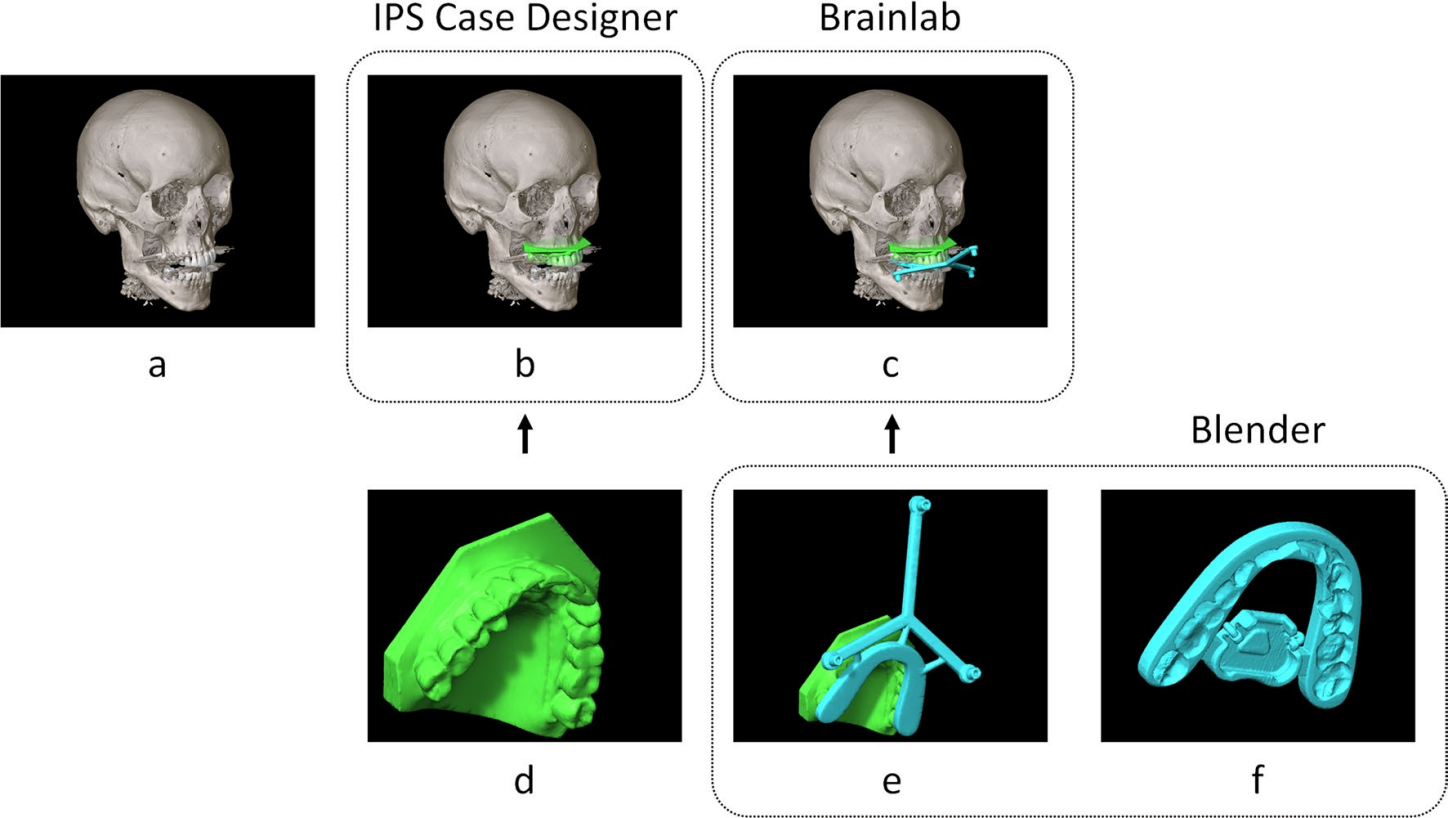
Workup of the registration-free workflow and software used. The CT scan (**a**) and intra-oral scan (**d**) are fused in IPS Case Designer (**b**). A splint-borne DRF is designed for optical tracking (**e**) and a splint-borne DRF holder is designed for EM tracking (**f**). After transformation, the DRF may be visualized in the Brainlab environment (**c**); the pose of the DRF in the image volume (linking patient space and image space) is established without a registration procedure.

The maxillary dentition was identified as a suitable anatomical structure to attach a DRF in a known and stable pose. An intra-oral scan of the maxillary dentition (TRIOS 3 intraoral scanner, 3Shape, Copenhagen, Denmark) was acquired to obtain a detailed virtual stereolithographic model (stl) of the dentition. The CT scan was imported in IPS CaseDesigner (version 1.4, KLS Martin, Tuttlingen, Germany) and the maxillary dental model was fused to the maxillary dentition of the CT scan^15^. The fused dental model in IPS was exported in stl format. In Blender (version 2.81, Blender Foundation, Amsterdam, The Netherlands), the maxillary dental model was imported and a splint fitting the dentition was designed. An offset of 0.1 mm for the dental model was used to ensure proper splint fit. Two augmentations of the splint were implemented to equip it with DRFs: one to equip the splint with reflective markers resembling the Skull Reference Array (Brainlab AG, Munich, Germany) for optical navigation, and one resembling the EM Reference Holder (Brainlab AG, Munich, Germany). Design of the splint took approximately 15–20 min. An outline of the registration-free workflow and a visualization of the designs are provided in Fig. 2. The designs were exported in stl format and manufactured through 3D printing with a PolyJet printer (Objet30 Prime, Stratasys Ltd., Eden Prairie, MN, USA). The designs were manufactured in transparent material (VeroClear). The geometries, configurations, and reference positions of the optical and EM DRF were provided by Brainlab.

The splint-borne DRF meets the prerequisite of a pre-operatively established pose of the DRF on the patient. The pose of the DRF in the image space should be established to link the patient space and image space. The transformation of the DRF to the position on the splint (in the IPS CaseDesigner image space, ${T}_{SPL\to IPS}$) was calculated in Blender. IPS CaseDesigner and Brainlab software construct their reference frames differently (voxel space and image space) and use a different image orientation (RAS and LPS). This means that an additional transformation is necessary to obtain the position of the splint-borne DRF in Brainlab image space (${T}_{SPL \to BL}$). The necessary information to obtain ${T}_{IPS\to BL}$ was extracted from the Image Position Patient (IPP) information in the DICOM header file of the CT scan (Appendix I).

When the splint-borne DRF is positioned during surgery, ${T}_{SPL\to BL}$ provides the link between patient space and image space and could thus be used as a substitute for the intra-operatively defined ${T}_{reg}$: the need for intra-operative registration is obsolete.

#### Registration-free workflow TRE assessment

Currently, the navigation software is not equipped with a functionality to set ${T}_{SPL\to BL}$ pre-operatively: a registration procedure is mandatory to use the navigation hardware. To circumvent this, a pre-registration procedure was performed (${T}_{reg}$), which is a temporary registration solely used as a workaround to meet the system’s demands. In the EM measurements, the splint with the DRF was positioned on the cadaver’s dentition and the pre-registration was performed using surface-based matching. In the optical navigation setting, the pre-registration was performed while the Skull Reference Array was fixated to the cadaver’s skull; point-based fiducial registration was used to determine ${T}_{reg}$. The Skull Reference Array was subsequently removed and replaced by the splint-borne DRF. In both tracking techniques, the splint was secured using power chains. The landmark positions ${\varvec{c}}$ were obtained similarly to bone-anchored fiducial registration and surface-based registration: five repetitions were performed by each observer (RS, FB) for each tracking technique (optical, EM). The splint was repositioned after each repetition, since this is the primary act that determines the measurement outcome.

**Figure 3:**
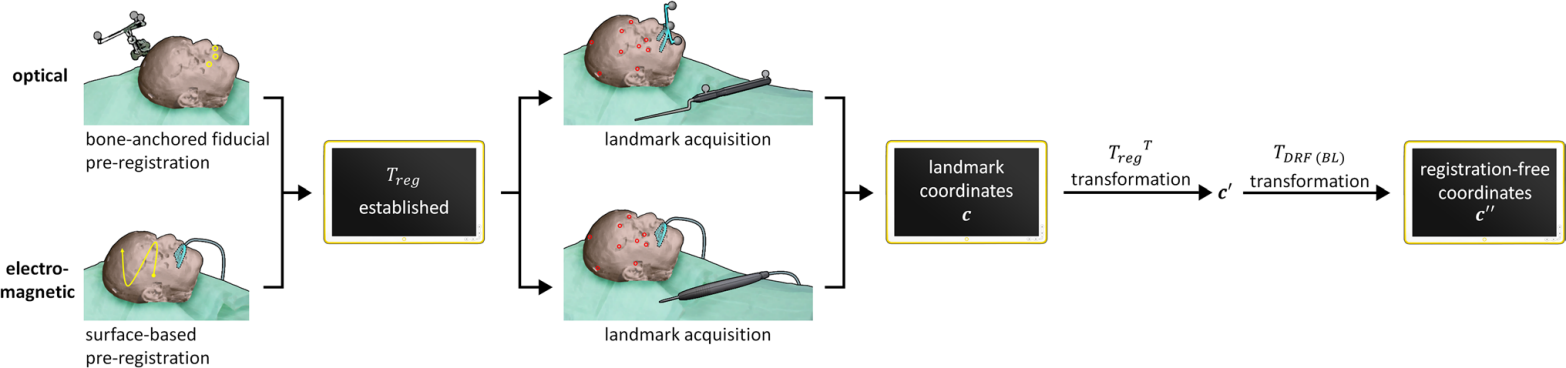
Flow chart of the use of transformations in the registration-free workflow. Two different overviews are presented: the method utilized in optical tracking is seen above, and the method in electromagnetic tracking is visualized below.

To assess the TRE in registration-free navigation, the following recalculation of the data was performed (Appendix I Fig. 2). First, the coordinate positions were transformed by ${{T}_{reg}}^{T}$, to correct the pre-registration. This yields an expression of the measured landmarks relative to the DRF: ${\varvec{c}}\boldsymbol{^{\prime}}$. Subsequently, the landmark coordinates were transformed by ${T}_{SPL\to BL}$, to obtain the measured coordinates of the registration-free navigation workflow in the image space (${\varvec{c}}\boldsymbol{^{\prime}}\boldsymbol{^{\prime}}$). These coordinates were compared to the actual positions of the landmarks in the image space (${\varvec{l}}$). A flow chart is presented in Fig. 3, which documents the use of the different transformations in the registration-free measurement process.

### Data processing

The data for each measurement session were stored in DICOM format and exported from the navigation system. In Matlab (version 2019b, the MathWorks Inc., Natick, MA, USA), the acquired landmarks were extracted from the DICOM data. The recalculation of the registration-free coordinates, as described in the subsection above, was performed in Matlab as well. The Euclidean distance between the resulting coordinate and the target coordinate was calculated (TRE); the Euclidean distances were exported as comma-separated values (csv). A linear mixed model incorporating all measurements was generated in R^16,17^. The fixed effects were tracking technique, registration method and target distance, as well as their interactions. The target distance was calculated as the distance of a fiducial to the centroid of the splint. The mean target distance of the infraorbital rim landmarks was calculated and subtracted from all target distances. This ensured that a clinically meaningful intercept was provided: the linear-mixed model outcome at distance = 0 represents the accuracy at the infraorbital rim.

## Results

**Figure 4:**
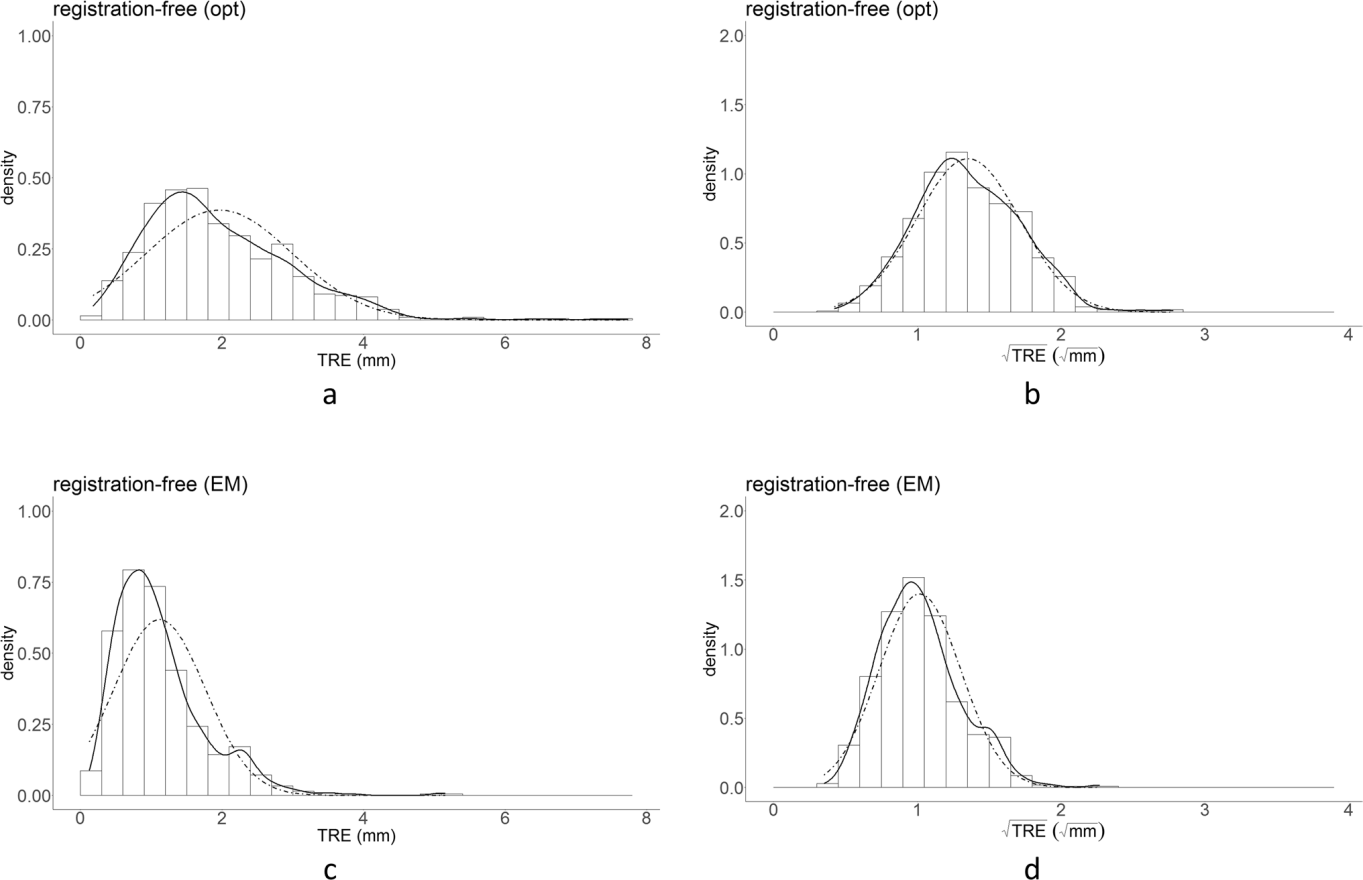
Distribution of TRE (**a**, **c**) and $\sqrt{TRE}$ (**b**, **d**) for the registration-free navigation approach. The dashed line represents a normal distribution with the mean and standard deviation of the outcome measure. From these histograms it is seen that the $\sqrt{TRE}$ distribution has a better resemblance to the normal distribution.

**Figure 5:**
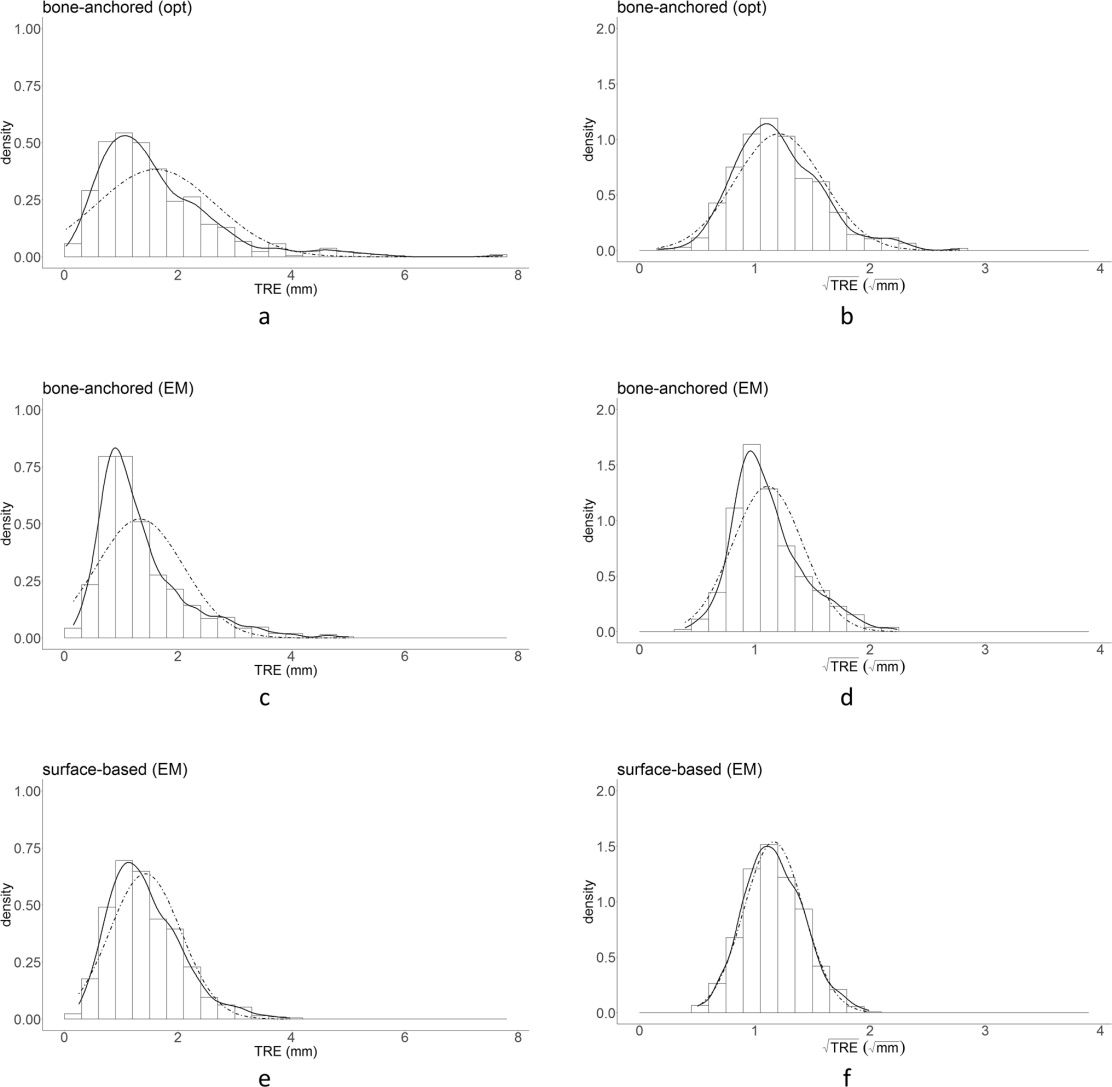
Distribution of TRE (**a**, **c**, **e**) and $\sqrt{TRE}$ (**b**, **d**, **f**) for bone-anchored optical, bone-anchored electromagnetic and soft-tissue registration respectively. Similar to Fig. 4, the dashed line represents a normal distribution. The $\sqrt{TRE}$ distribution also has a better resemblance to the normal distribution for these registration approaches.

**Figure 6:**
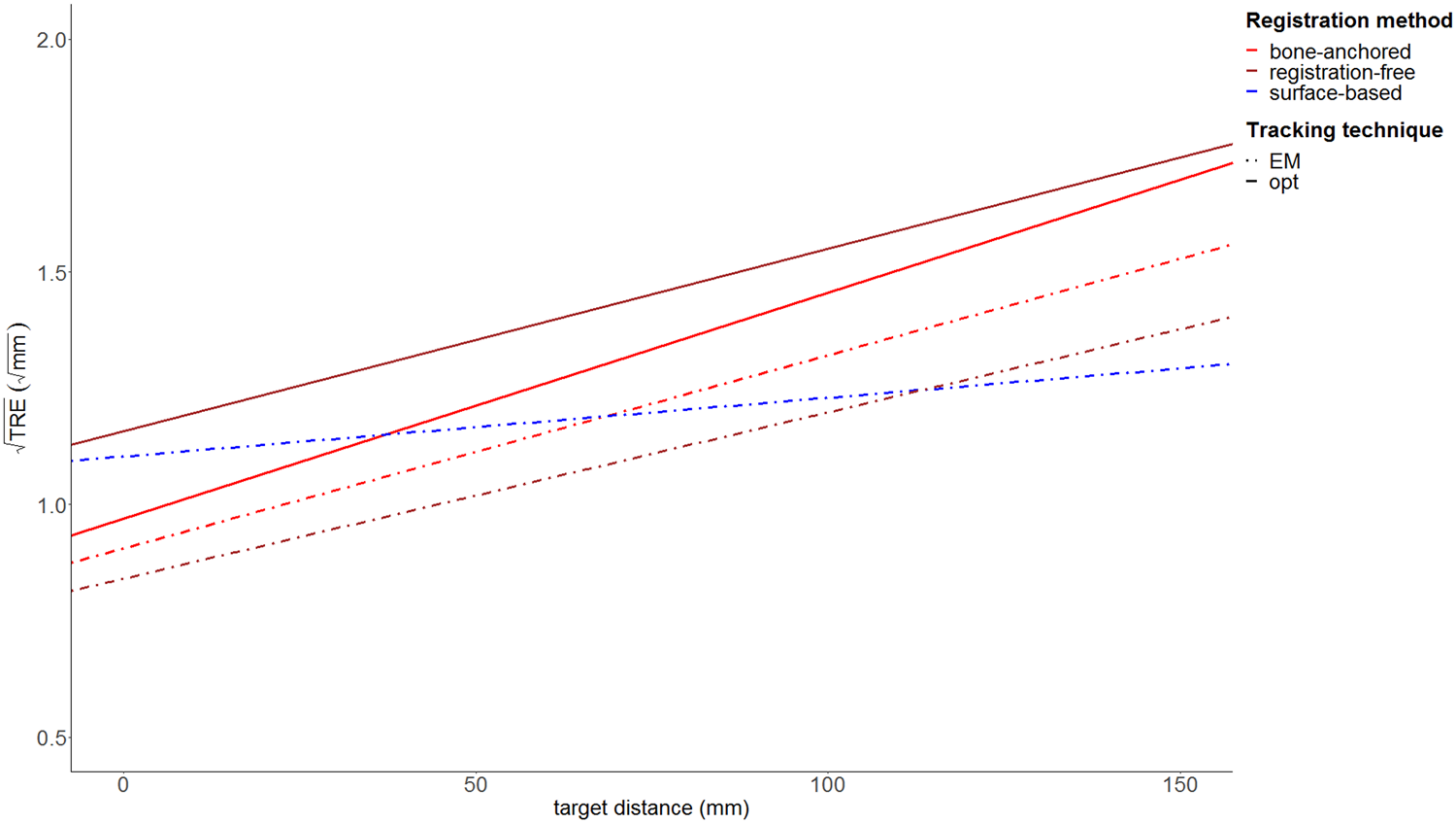
Visualization of the regression lines for each registration method and tracking technique. The brown lines show the registration-free technique.

1396 measurements were obtained using registration-free navigation, 4 (0.3%) were missing (1 registration method * 2 tracking systems * 2 observers * 5 repetitions * 5 cadavers * 14 target points—4). In total, 3496 data points are included in the results (1396 registration-free (2 tracking methods), 1400 bone-anchored fiducials (2 tracking methods), 700 soft-tissue registration (electromagnetic tracking)). In Fig. 4, histograms and kernel density estimates for the TRE and $\sqrt{TRE}$ are provided for registration-free navigation and in Fig. 5 the histograms and kernel density estimates for bone-anchored fiducials and soft-tissue registration. The $\sqrt{TRE}$ data distributions most closely represent a normal distribution. In Table 2, the fixed effect estimate output of the complete linear mixed model is provided. The data are recalculated to an intercept value (at the level of the infraorbital rim) and a slope value (increase $\sqrt{TRE}$ per mm distance from the intercept) for each combination of registration method and tracking technique in Table 3. The bold font in Tables 2 and 3 indicates the results for registration-free navigation. In Fig. 6, $\sqrt{\mathrm{TRE}}$ is plotted against target distance for all combinations, by using the acquired slope and intercept values.

**Table 2:** Fixed effect estimates. Bone-anchored registration with optical tracking was the model’s reference category. The bold font are the registration-free parameters.

Fixed effect	Estimate	Standard dev	t value
(Intercept)	0.970	0.0348	27.86
Distance	+ 0.005	0.0002	23.07
EM technique	− 0.064	0.0202	− 3.19
**Registration-free**	** + 0.188**	**0.0202**	**9.31**
Surface-based registration	+ 0.197	0.0202	9.78
Distance:EM technique	− 0.001	0.0003	− 2.35
**Distance:Registration-free**	** − 0.001**	**0.0003**	** − 3.13**
Distance:Surface-based registration	− 0.003	0.0003	− 9.73
**EM technique:Registration-free**	** − 0.253**	**0.0286**	** − 8.87**
**Distance:EM technique:Registration-free**	** + 0.000**	**0.0004**	**0.86**

**Table 3:** Intercept and slope values from linear mixed model parameters.

Tracking technique	Registration method	Intercept	Slope
Optical	Bone-anchored fiducials	0.97	0.0049
**Optical**	**Registration-free**	**1.16**	**0.0039**
Electromagnetic	Bone anchored fiducials	0.91	0.0042
Electromagnetic	Surface-based	1.10	0.0013
**Electromagnetic**	**Registration-free**	**0.84**	**0.0036**

**Figure 7:**
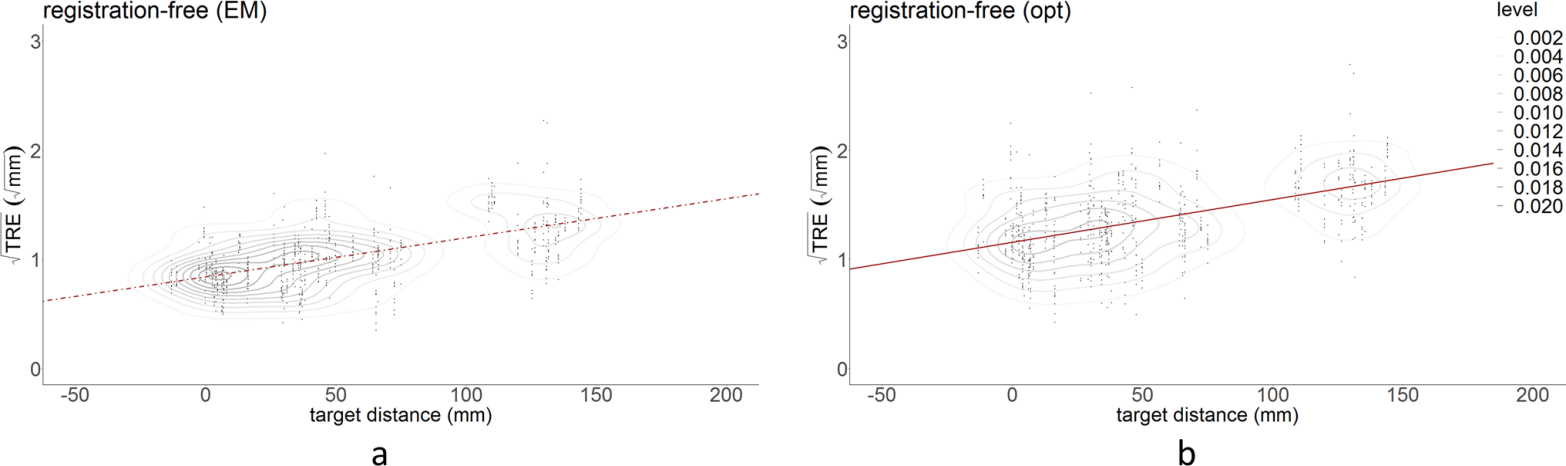
Scatter plots, with kernel density estimate levels for the registration-free workflow in electromagnetic (**a**) and optical (**b**) tracking. The regression lines from Fig. 4 are superimposed. The EM measurements are less scattered than the optical measurements.

The plot in Fig. 6 demonstrates that the EM registration-free approach outperforms all other registration approaches, but the optical registration-free approach is outperformed by bone-anchored fiducial optical registration and all EM registration approaches. In Fig. 7, combined kernel density estimate and scatter plots are given for the registration-free data. The regression lines from Fig. 6 are superimposed on the scatter plots. For the EM registration-free measurements, 11% of TRE measurements was > 2 mm, for the optical measurements this was 41% (compared to 27% for optical bone-anchored fiducials, 15% for electromagnetic bone-anchored fiducials and 17% for electromagnetic soft-tissue registration).

## Discussion

A novel registration-free approach for orbitocraniofacial intra-operative navigation was introduced in this study. The target accuracy of the method was evaluated and compared to the accuracy of bone-anchored fiducial registration and surface-based registration. Bone-anchored fiducial registration proved more accurate at the infraorbital rim, but was more heavily affected by increasing target distances than soft-tissue registration. The increased accuracy at the infraorbital rim may be explained by the proximity of this landmark to the registration fiducials. The limited variation in craniocaudal direction of the fiducials gives rise to a relative coplanar orientation, which is known to yield increasing TRE values moving away from the registration centroid^18^. The configuration of these fiducials was chosen to mimic the clinical setting as closely as possible, rather than aim for the optimal TRE value. The registration-free approach yielded excellent results compared to conventional approaches with EM tracking, but the results in the optical tracking setting were unfavorable to any of the alternative intra-operative registration methods. This large deviation between the tracking methods makes the results difficult to interpret. The deviation in results between both tracking techniques is unexpected Two differences can be distinguished between the registration-free procedure for EM and optical navigation: one regarding design and one regarding pre-registration procedure. These differences and their possible effect on the registration accuracy will be detailed below, but neither provides a solid explanation for the difference or its magnitude.

The attachment of the DRF on the registration splint is different between the optical and electromagnetic setting. The EM tracker is positioned within a holder on the palatal side of the splint; the holder is attached to the molar region on either side. The optical tracker is not attached to the design but is incorporated in it: the reflective markers are attached to three arms extending on the mesial side of the central incisors. While direct incorporation in the design should be less error-prone, the optical design may suffer from reduced stiffness in the material because of the extensions’ length, which might have affected the true position of the reflective marker spheres and thus the DRF as a whole. Venosta et al. noticed the influence of material stiffness on registration accuracy in their extended splint design^5^. The DRF splint was designed bearing maximum bending resistance in mind: a rounded edge design was chosen for the extensions over a cubic one for precisely this reason. The location of the DRF in relation to the dentition might also have influenced positioning accuracy. After securing the splint, positional deviation between the DRF in EM may be mostly due to differences in splint fit on the molars, while a positional deviation in the optical tracker may occur because of a difference in fit between the molar region and the incisors. Ye et al. have investigated splint fit, and in their results a difference in splint fit between the incisor region and the molars can be seen^19^. However, this difference is only minimal and the recommended offset of 0.1 mm, which resulted in the smallest fit deviation, was used in this study.

The second difference was in the mandatory pre-registration (performed to meet the requirements of the navigation system, but corrected for by the back-transformation). As stated in the methods section, the EM DRF was positioned on the splint during pre-registration (with surface-based matching) while the optical skull-fixated optical DRF was exchanged for the splint-borne DRF. This workflow was chosen because surface-based matching was not available in the optical setting, and the position of the splint DRF would interfere with the registration process on the bone-anchored fiducials. The landmarks collected with the Acquire-functionality were outside the image volume assigned in the DICOM information in the optical tracking, but their coordinates were still registered. The pre-registration transformation, and any error associated with it, was corrected in a similar fashion for optical and electromagnetic tracking, so this should not have had any influence on the registration-free TRE measurements. The orientation difference between the skull-fixated and splint-borne dynamic reference frames might give rise to some technical error in measuring the positions of the reflective marker spheres by the optical camera, but this error is not expected to be in the order of magnitude of the TRE difference. A final explanation might be an inaccuracy in the design or fabrication of the optical splint-borne optical DRF. The virtual designs were checked and the geometry of the optical spheres was controlled with distance measurements. No substantial errors were found using these methods and all splint-borne DRFs were recognized by the navigation system; any significant deviation in design or manufacturing would have precluded this.

Several studies have proposed designs of a Dynamic Reference Frame supported by a splint^20–31^. This position of the Dynamic Reference Frame may be less invasive than fixation on the patient’s cranium^32^. Registration outside the patient has been proposed, both with a fiducial-based^20–23,25–27^ and automatic method^28–31^. The fiducial-based registration is possible if the design has both registration fiducials and the DRF attached to the splint. The splint and the fiducials need to be positioned in the patient’s mouth during acquisition of the scan; the DRF may be rigidly fixated to the splint or attached later. The relationship between the fiducials and the DRF will not change, so registration can be performed before the splint is positioned in the patient’s mouth. A prerequisite is that the splint position does not differ between image acquisition and surgical setting. This workflow is still susceptible to Fiducial Localization Errors in the image volume and physical space, since a registration still needs to be performed^4^. In the automatic registration methods proposed, the DRF is connected to the splint and present in the image volume, or a unique connection between splint and DRF is designed. The pose of the DRF in the image volume may be determined on the pre-operative scan. This method is similar to the registration process described here, but requires image acquisition with the splint in position, which frequently leads to acquisition of a second scan and additional radiation exposure for the patient.

Intra-operative Automatic Image Registration suffers from the same drawback. In this workflow, the Dynamic Reference Frame is fixated intra-operatively, and during acquisition of a Cone-Beam CT (CBCT) the DRF is tracked with the optical camera^7,10,12,33^. This method yields an accurate registration, even if low-dose scan protocols are used, but may lead to an increase in operation time and pose logistical challenges intra-operatively^10,12^. A compatible intra-operative scanner is required, and currently, this method is only available for optical tracking. Other methods of user-independent registration have been proposed: a stereotactic mask, which uses active LEDs, or 3D stereophotogrammetry to capture the soft-tissue of the patient with the DRF in place^9,34–36^. The stereotactic mask is attached to the patient’s face and is used for both registration and tracking, which means that the mask has to stay attached during the complete procedure. This may limit its application in reconstructive surgery of the midface^9,35^. With 3D stereophotogrammetry, the soft-tissue surface is captured through (3D) photographs of the patient with the DRF in place^34–36^. A large surface of the skin needs to be exposed for the photographs and methods relying on soft-tissues are susceptible to skin surface alterations. These methods may thus not be applicable in situations where soft-tissue variation is to be expected (e.g. swelling, nasal intubation)^9,36^.

The dentition may be used as a reference in a direct or indirect way in Augmented Reality (AR)^37–39^. Wang et al. designed a method in which an intra-oral scan is matched to the CT scan (based on an Iterative Closest Point approach)^39^. The visible teeth of the patient are tracked with a stereo camera. After registration of the stereo camera images with the intra-oral scan model, the physical world can be augmented with the virtual planning. Exposure of the dentition, within the field-of-view, is a requirement for the workflow. Jiang et al. have proposed an AR workflow resembling the registration-free workflow described in this study^37^. An intra-oral scan of the gypsum cast with the DRF in place is acquired, and the resulting model is matched to the CT scan using user-indicated landmarks on the dental cusps in the CT model and intra-oral scan. In the workflow described in the current study, the DRF is not positioned during the intra-oral scan, which allows an intra-oral scan of the complete dentition. Moreover, the algorithm that matches the dental model on the CT scan is not user dependent. These differences in approach may lead to an improvement in matching accuracy of the intra-oral scan^40^, but might lack control of the splint fit on the dentition.

The registration-free navigation workflow is a method that is non-invasive and not user dependent. It is compatible with both optical and electromagnetic navigation. It could lead to a more time-efficient intra-operative procedure since intra-operative registration is obviated. This requires that the workflow is implemented in the commercialized navigation system, so that the registration matrix is determined directly rather than necessarily correcting a pre-registration as was done in this study. The workflow may be used in (partly) dentate patients whose maxillary complex is intact and continuous with the cranium. Fixation of the splint without affecting the non-invasive character of the method should be improved. Ideally, the splint would snap in place on the dentition. The method proved accurate if electromagnetic tracking was used. In contrast, a large error was found if optical tracking was used. No clear explanation for the difference between optical and electromagnetic navigation was found, although the accuracy in the optical navigation setting might improve to some extent if material stiffness and splint design are optimized. Thus, the biggest challenge toward clinical implementation of the workflow lies in reducing the error in optical navigation.

## Conclusion

A registration-free workflow for optical and electromagnetic craniofacial intra-operative navigation was presented in this study. This method offers a non-invasive, user-independent alternative to existing registration procedures and may thus lead to increased time efficiency intra-operatively. The accuracy of the method was evaluated on five human cadaver heads; the results were compared to the accuracy measurements of maxillary bone-anchored fiducials (optical and electromagnetic) and surface-based registration (electromagnetic). The accuracy for optical and electromagnetic tracking differs greatly: registration-free navigation in electromagnetic tracking showed very promising results, while registration-free optical navigation was the least accurate of all methods. Although the workflow itself is promising, this difference in results, without a valid explanation, hampers direct clinical implementation.

## Supplementary Information

Supplementary Information.

## Data Availability

The datasets generated during and/or analysed during the current study are available from the corresponding author on reasonable request.
