# Supplementary material for: Registration-free workflow for electromagnetic and optical navigation in orbital and craniofacial surgery
Source: Sci Rep. 2021 Sep 10;11:18080. doi: 10.1038/s41598-021-97706-5 (PMC8433137; doi:10.1038/s41598-021-97706-5)
Supplement: Supplementary file 1 — Supplementary Information. [file 41598_2021_97706_MOESM1_ESM.docx]

**APPENDIX I**

Overview of transformations and coordinates

*Theoretical background*

Patient space: physical space (in the operating room).

Image space: virtual space made up by the patient’s image volume.

$T_{DRF}$: transformation of Dynamic Reference Frame from the origin of the world coordinate system to its pose on the patient (patient space). Transformed to image space with $T_{reg}$.

$T_{PTR}$: pose of the navigation pointer instrument in the patient space. $t_{PTR}$ is the translation component of $T_{PTR}$.

$T_{DRF\to PTR}$: transformation between DRF position and pointer position in the patient space.

$T_{reg}$: solution to the rigid-body registration equations, obtained with the registration procedure. It provides an approximation of $T_{DRF}$’s position in the image space: it links patient space and image space. $T_{reg}$ is stored in the DICOM header information by the navigation system (and may be extracted).

*Practical implementation*

$T_{SPL\to IPS}$: pose of the DRF on the splint in the image space of the IPS software. This transformation can be extracted (preoperatively) from the splint design in the Blender environment.

$T_{IPS\to BL}$: transformation used to transform objects (in this case, the spint-borne DRF) from the IPS image space to the Brainlab image space. The information to construct this transformation is extracted from the DICOM header tag (0020,0032) (ImagePositionPatient, IPP) of the preoperative CT scan:

$$T_{IPS\to BL}=\left[ \begin{matrix} -1 & 0 & 0 & -X_{IPP} \\ 0 & -1 & 0 & -Y_{IPP} \\ 0 & 0 & 1 & Z_{IPP} \\ 0 & 0 & 0 & 1 \end{matrix} \right]$$

$T_{SPL\to BL}$: pose of the DRF in the Brainlab image space. This transformation is used as a pre-operatively defined approximation of $T_{DRF}$ in the registration-free workflow.

*Registration-free TRE measurement*

$l$: true coordinates of a landmark position on a PEEK screw (see Methods – Preparations) in the image space.

$c$: measured coordinates in the image space if the navigation pointer is positioned at a landmark position.

$c'$: coordinates of $c$, corrected for pre-registration (DRF positioned at the origin of the world coordinate system).

$c''$: measured coordinates of $l$ in the registration-free workflow in the image space.

N.B. bold notation indicates a vector of all landmark coordinates.

Schematic drawings


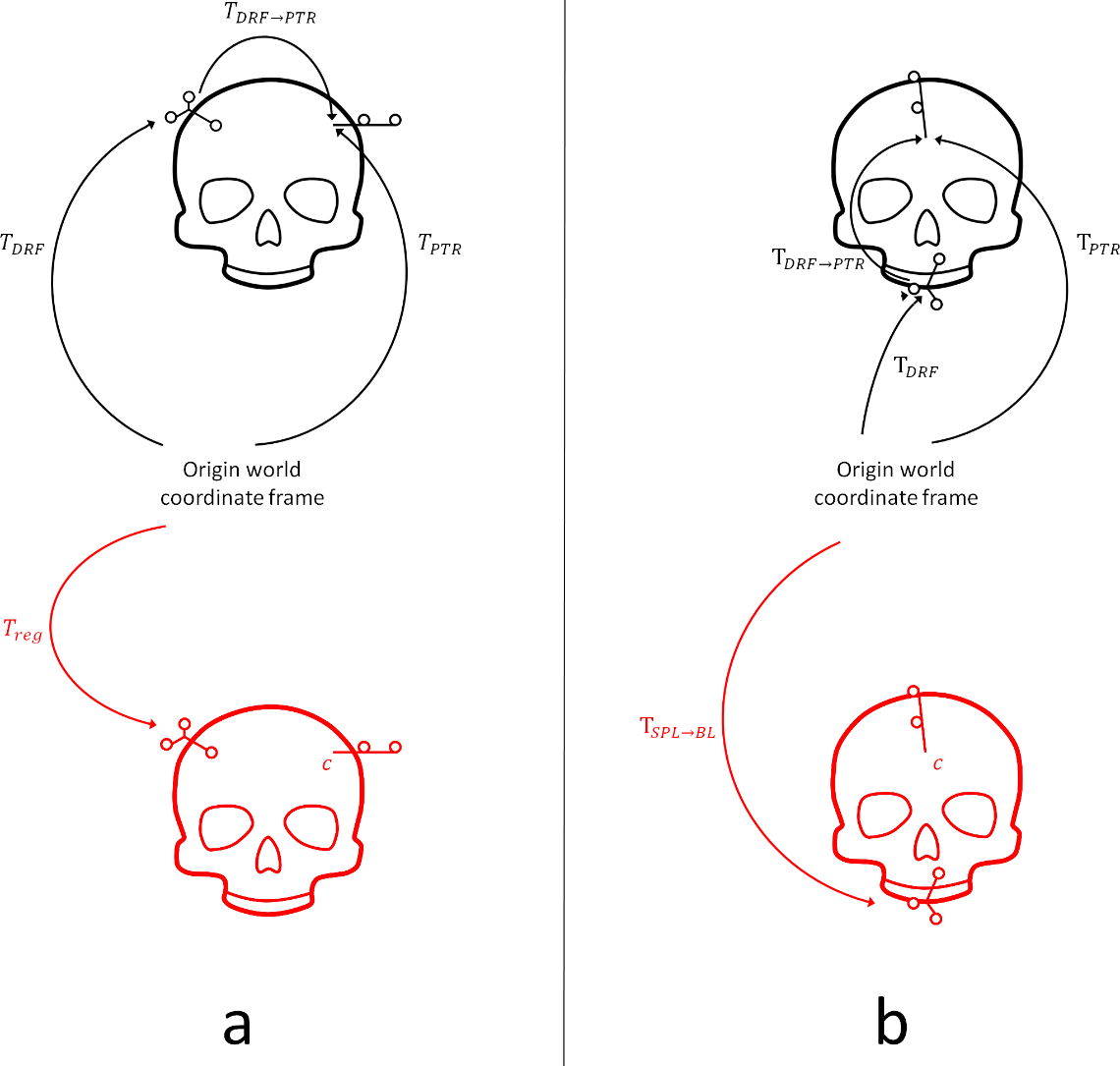


*Appendix I Figure I. Theoretical background of registration-free navigation. Navigation with a regular registration procedure is shown in* ***a****, registration-free navigation is shown in* ***b****; black refers to the patient space (physical space) and red to the image space. In* ***a****, the DRF is attached to the patient’s skull (*$T_{DRF}$*) and* $T_{PTR}$ *the pose of the navigation pointer (with position* $t_{PTR}$*);* $T_{DRF\to PTR}$ *is the transformation between pointer and instrument.* $T_{reg}$*, obtained with a registration procedure, links the physical space and image space. After registration, coordinates* $c$ *are the position of the pointer in the image space. In registration-free navigation, the DRF is attached to a splint and positioned on the patient’s dentition in the patient space (*$T_{DRF}$*); the position of the DRF in the image space is provided by* $T_{SPL\to BL}$*; the physical space and image space are linked without a registration procedure.*


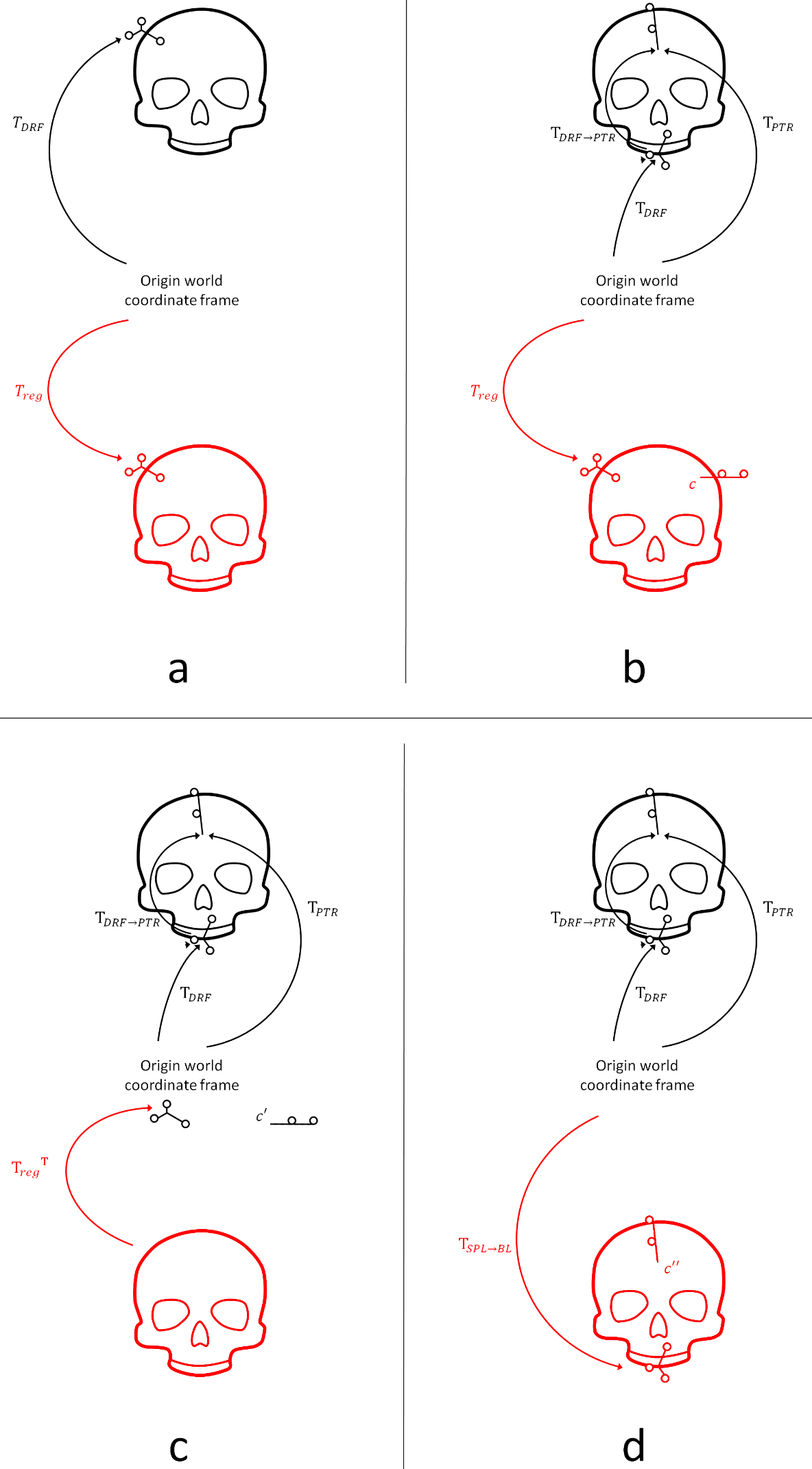


*Appendix I Figure II. Registration-free TRE measurement. This is a workaround, since it is not feasible to set* $T_{SPL\to BL}$ *in the navigation equipment. In Figure* ***a****, a pre-registration (*$T_{reg}$*, corrected later) is performed with the DRF attached to the lateral skull in the patient space (*$T_{DRF}$*). In Figure* ***b****, the skull-fixated DRF is exchanged for the splint-borne DRF. During the TRE measurements, the pointer is positioned at the PEEK screw (patient space). The navigation system will acquire the coordinates* $c$*, from* $T_{reg}$ *and* $T_{DRF\to PTR}$*; it can be seen that* $c$ *does not match the pointer’s position in the patient space since the DRF has been moved. In Figure* ***c****, a transformation with* ${T_{reg}}^{T}$ *is performed to the acquired coordinates* $c$ *to obtain* $c'$*: coordinates with the DRF positioned at the origin of the world coordinate frame.. In the final stage shown in Figure* ***d****,* $c'$ *is transformed by* $T_{SPL\to BL}$*, to obtain coordinates* $c''$ *in the image space. These coordinates in the image space match the position of the pointer in the patient space. The TRE is calculated from the difference between* $l$ *and* $c^{''}$*.*

Equations

$${T_{SPL\to BL}=T_{SPL\to IPS} T}_{IPS\to BL}$$

$$\boldsymbol{c}^{\boldsymbol{'}}={T_{reg}}^{T}\boldsymbol{c}$$

$$\boldsymbol{c}^{\boldsymbol{''}}=T_{SPL\to BL}\boldsymbol{c}\boldsymbol{'}$$
